# Spent Coffee Grounds and Novaluron Are Toxic to *Aedes aegypti* (Diptera: Culicidae) Larvae

**DOI:** 10.3390/insects14060564

**Published:** 2023-06-16

**Authors:** Waralee Thanasoponkul, Tanasak Changbunjong, Rattanavadee Sukkurd, Tawee Saiwichai

**Affiliations:** 1 Department of Parasitology and Entomology, Faculty of Public Health, Mahidol University, Bangkok 10400, Thailand; waralee.tha@mahidol.ac.th (W.T.); rattanavadee.suk@mahidol.ac.th (R.S.); 2 Department of Pre-Clinic and Applied Animal Science, Faculty of Veterinary Science, Mahidol University, Nakhon Pathom 73170, Thailand; tanasak.cha@mahidol.edu; 3 The Monitoring and Surveillance Center for Zoonotic Diseases in Wildlife and Exotic Animals (MoZWE), Faculty of Veterinary Science, Mahidol University, Nakhon Pathom 73170, Thailand

**Keywords:** coffee grounds, health, insect growth regulator, larvicide, mosquito control, synergistic effect

## Abstract

**Simple Summary:**

The mosquito *Aedes aegypti* has developed insecticide resistance in Thailand and around the world. We investigated the effective forms between wet and dry spent coffee grounds (wSCGs and dSCGs) and novaluron on larval mortality and adult emergence inhibition of *Ae. aegypti*. The main chemical compound concentration in wSCGs was higher than that in dSCGs. Then, the effective SCGs were selected to combine with novaluron and determined the synergistic effects of its combination. At sublethal concentrations wSCGs and novaluron had low efficacies when present individually; however, when combined at low concentrations, they showed greater efficacy. Therefore, wSCGs combined with novaluron could be utilized as an alternative control for this mosquito vector.

**Abstract:**

*Aedes aegypti* (Diptera: Culicidae) is a vector for mosquito-borne diseases worldwide. Insecticide resistance is a major concern in controlling this mosquito. We investigated the chemical compounds in wet and dry spent coffee grounds (wSCGs and dSCGs) and evaluated the efficacy of dSCGs, wSCGs, and novaluron on the mortality and adult emergence inhibition of *Ae. aegypti*. We found higher concentrations of chemical compounds in wSCGs than in dSCGs. The wSCGs and dSCGs both contained total phenolic compounds, total flavonoid compounds, caffeic acid, coumaric acid, protocatechuic acid, and vanillic acid. Complete mortality was observed after 48 h of exposure to 50 g/L wSCGs, while similar mortality was found after 120 h of exposure to 10 µg/L of novaluron. The sublethal dose was a concentration of wSCGs (5 g/L) and novaluron (0.01, 0.1, and 1 µg/L) combined that resulted in a larval mortality lower than twenty percent (at 72 h) to determine their synergistic effects. The death rate of larvae exposed in sublethal combination of wSCGs and novaluron was significantly higher than that of its stand-alone. The findings indicate that the combination of wSCGs and novaluron at sublethal concentrations had synergistic effects on the mortality of *Ae. aegypti* larvae and could be applied as an alternative control measure.

## 1. Introduction

Vector-borne diseases (VBDs) are infectious diseases transmitted by vectors. Dengue is one of the prominent VBDs that affects public health, causing more than 700,000 deaths annually [1]. *Aedes aegypti* is the primary vector of dengue while *Ae. albopictus* plays the role as secondary vector [2,3]. The best way to prevent and control dengue fever is controlling *Ae. aegypti* mosquitoes. Increasing chemical resistance and environmental residues are major concerns affecting the chemical control of mosquitoes. Resistance of *Ae. aegypti* to synthetic insecticides has been reported in many countries worldwide. Temephos is an example of larvicide to which larval resistance is found in countries such as Thailand [4,5,6], Colombia [7], Peru [8], and Ecuador [9]. The use of herbal or natural substances for alternative mosquito control is a popular strategy that has gained attention worldwide.

Coffee is one of the most popular beverages worldwide, with more than 1.4 billion cups consumed daily. Consequently, more than 15 million tons of spent coffee grounds (SCGs) are left to waste each year [10]. SCGs have been reused for various applications, such as food ingredients [11], renewable energy [12], soil improvement [13], and mosquito control [14,15]. Mortality of *Ae. aegypti* larvae exposed to 300 mg/mL SCGs has been reported at 100% [14]. Furthermore, 50 and 100 mg/mL of SCG concentrations inhibited larval development of *Ae. aegypti*, causing complete death of the larvae in the L3 and L2 stages, respectively [15]. Furthermore, arabica coffee grounds have been found to be lethal to third-instar larvae of *Ae. aegypti* with an LC_50_ of 33.66 g/L [16]. 

Novaluron, (±)-1-[3-chloro-4-(1,1,2-trifluoro-2-trifluoro-methoxyethoxy) phenyl]-3-(2,6-difluorobenzoyl)urea, is an insect growth regulator (IGR) insecticide. It acts as a chitin synthesis inhibitor with the active agent being the benzoylphenyl urea (BPU) group [17]. The BPU group inhibits *N*-acetylglucosamine production in chitin synthesis in vivo [18,19] and is active during the egg and larval stages, causing larval death or the development of incomplete adults [19]. Novaluron acts on insects via ingestion or direct contact, and thus is commonly used in agriculture [20]. The World Health Organization Pesticide Evaluation Scheme (WHOPES) has suggested that novaluron can be used as a mosquito larvicide, with a recommended dosage of 10–50 µg/L [21]. Novaluron has very low toxicity for rats, birds, earthworms and honey bees [20]. Furthermore, novaluron is non-genotoxic and non-carcinogenic to humans, is non-toxic to developing systems, and is non-mutagenic [20].

Chemical resistance can be reduced in mosquito control by several ways, such as the rotation of chemicals use, the use of natural substances, or a combination of chemicals and natural substances. We hypothesized that wSCGs are more toxic to *Aedes* larvae than dSCGs and also effective when combined with novaluron. Therefore, this study aimed to investigate the chemical compounds in wet and dry forms of SCGs (wSCGs and dSCGs) and evaluate the efficacy of wSCGs, dSCGs, and novaluron on larval mortality and adult emergence inhibition of *Ae. aegypti*. Thus, sublethal concentrations of better efficacy forms of SCGs and novaluron were combined to determine their synergistic effects. 

## 2. Materials and Methods

### 2.1. Mosquitoes

Eggs of *Ae. aegypti* (THAI NIH, Laboratory strain) were provided by the Insect Taxonomy and Reference Museum Section and Entomological Support Section, Medical Entomology Group, National Institute of Health, Department of Medical Sciences, Ministry of Public Health, Thailand. Mosquitoes were maintained in the Department of Parasitology and Entomology Laboratory, Faculty of Public Health, Mahidol University, at a temperature of 25 ± 3 °C, relative humidity of 70 ± 20%, and photoperiod of 12L:12D. Before starting the bioassay, the eggs of *Ae. aegypti* will be reared until they developed into the third-instar larvae, which were used for the bioassay. The protocol for this study was approved by the Mahidol University Center of Ethical Reinforcement for Research (MUCERR) (reference no. MU-IACUC 2023/013). Biosafety in laboratories was approved by the Mahidol University-Institutional Biosafety Committee (MU-IBC), Faculty of Public Health, Mahidol University (reference no. FPH-BS 005-2022).

### 2.2. Spent Coffee Grounds

Roasted arabica coffee beans originating from Mae Sot District, Tak Province, Thailand were purchased from Doi Muser coffee shop (Tambon Dan Mae Lamoh, Mae Sot District). To prepare the SCGs, 10 g of crushed coffee beans was dripped using a drip coffee bag and 150 mL of hot water (92–96 °C) was added. wSCGs were then used immediately. dSCGs were dried in a hot air oven at 60 °C for 16 h. Five concentrations (0 (control), 5, 10, 25, and 50 g/L) of dry weight equilibrium of wSCGs and dSCGs were prepared for bioassay.

The concentrations of chemical compounds in the wSCGs and dSCGs were analyzed by triple readings in each sample of three replicates as follows. Total phenolic compounds were determined using a microplate reader (Biotek PowerWave XS; Agilent Technologies, Santa Clara, CA, USA) according to the Folin–Ciocalteu procedure [22]. The total flavonoid compounds were determined by aluminum chloride assay using a spectrophotometer (Shimadzu UV-2550; SHIMADZU, Tokyo, Japan) [23]. High-performance liquid chromatography was used to evaluate compounds, such as caffeic acid, coumaric acid, ferulic acid, gallic acid, protocatechuic acid, sinapic acid, vanillic acid, and caffeine as described previously [24]. The concentrations were calculated based on dry weight.

### 2.3. Novaluron

Novaluron (Novaluron, Dr. Ehrenstorfer™, Purity 98.77% (g/g), Product Code DRE-C15653000, Lot no. G1101723) was used in the experiment. The stock solution was prepared by dissolving 50 mg novaluron in 1000 µL acetone. A 10-fold serial dilution was then made for working concentrations. The bioassay was evaluated using five concentrations (0 (control), 0.01, 0.1, 1, and 10 µg/L) of novaluron in dechlorinated water.

### 2.4. Bioassay

Larval mortality was investigated using the third-instar larvae of *Ae. aegypti* (30 larvae/cup) exposed in 300 mL of different concentrations of novaluron and SCGs with four replicates, as previously described [16]. In brief, the larvae were exposed to wSCGs, dSCGs, and novaluron individually and in combinations in the given concentrations, and the mortality was observed at 6, 12, 24, 48, 72, 96, and 120 h of exposure. To prevent the pooled infusions of larvae and SCGs, which resulted in difficulty for larva counting, the mixtures were sieved with fine mesh mosquito net. The suspended SCGs were separated to the bottom of the test cup by a net, and the larvae were exposed to the upper half of the test cup. During the experiment, the larvae were fed with fish food pellets. After the adults emerged, they were fed with 10% sucrose solution in cotton balls.

### 2.5. Combination of wSCGs and Novaluron

After the bioassay for the efficacy of wSCGs and novaluron on larval mortality at 72 h, sublethal concentrations of wSCGs and novaluron that caused mortality lower than twenty percent were selected and combined to investigate their synergistic effects. We selected 5 g/L wSCGs to combine with three concentrations of novaluron (0.01, 0.1, and 1 µg/L) for the bioassay.

### 2.6. Statistical Analysis

The percentage of larval mortality and adult emergence inhibition was presented as the mean ± standard deviation. The experiments were stopped and repeated when the mortality in the control group exceeded 20%. The mortality was corrected using Abbott’s formula when the mortality in the control group was >5% [25]. The larval mortality and adult emergence inhibition rates between the groups of stand-alone and combination treatments were analyzed using the Mann–Whitney U test. In addition, the Kruskal–Wallis test was employed to compare the larval mortality and adult emergence inhibition rates across all stand-alone and combination treatments. A significance level of *p* < 0.05 was considered statistically significant. The synergistic effect was determined by measuring the larval mortality in the sublethal combination treatment, which was higher than that of its stand-alone treatment.

## 3. Results

### 3.1. Characteristics and Quantification of wSCGs and dSCGs

The wSCGs and dSCGs were dark brown and brown in color, respectively. The weight ratio of dSCGs to wSCGs was 1.0:3.2 g. The chemical analysis identified total phenolics, four phenolic compounds, total flavonoids, and caffeine (an alkaloid) in the wSCGs and dSCGs (Table 1). The predominant phenolic compounds in wSCGs included vanillic acid (5597.0 ± 213.8 µg/g) and caffeic acid (329.7 ± 0.4 µg/g), whereas vanillic acid (770.3 ± 22.5 µg/g) and protocatechuic acid (9.1 ± 3.0 µg/g) were the predominant compounds in dSCGs. The concentrations of ferulic acid, gallic acid, or sinapic acid were undetectable in either wSCGs or dSCGs (Table 1).

### 3.2. Comparison of Larval Mortality and Adult Emergence Inhibition with wSCGs and dSCGs

The death of larvae exposed to wSCGs began after 6 h, but there were no larval deaths in the dSCGs exposure groups. All larvae exposed to wSCG at 50 g/L died completely after 48 h. At 72 h, the 0 (control), 5 (wSCG 5), 10 (wSCG 10), 25 (wSCG 25), and 50 (wSCG 50) g/L wSCG treatment groups had mortalities of 0.8 ± 1.7%, 16.7 ± 2.7%, 24.2 ± 3.2%, 54.2 ± 3.2%, and 100.0%, respectively. However, dSCGs at the same exposure concentrations had mortalities of 0.8 ± 1.7%, 8.3 ± 1.9%, 10.8 ± 3.2%, 14.2 ± 4.2%, and 14.2 ± 5.0%, respectively. Therefore, wSCGs were found to be more effective at causing mortality than that of the dSCGs. The larval mortality rates were recorded at 72 h for different concentrations of wSCGs and dSCGs were statistically significant (*p* < 0.05), as shown in Table 2.

The adult emergence inhibition of the *Ae. aegypti* larvae with the wSCGs was more effective than that with the dSCGs. At 120 h, the control, wSCG 5, wSCG 10, wSCG 25, and wSCG 50 had adult emergence inhibition rates of 0.8 ± 1.7%, 31.7 ± 6.4%, 40.8 ± 5.7%, 72.5 ± 5.0%, and 100.0%, respectively. The control, dSCG 5, dSCG 10, dSCG 25, and dSCG 50 had adult emergence inhibition rates of 0.8 ± 1.7%, 14.2 ± 3.2%, 16.7 ± 6.7%, 24.2 ± 1.7%, and 25.0 ± 7.9%, respectively. The adult emergence inhibition rates were recorded at 120 h for different concentrations of wSCGs and dSCGs were statistically significant (*p* < 0.05), as shown in Table 3.

### 3.3. Bioassay of Individual and Combination of wSCGs and Novaluron

#### 3.3.1. Individual Efficacies of wSCGs and Novaluron

**Figure 1:**
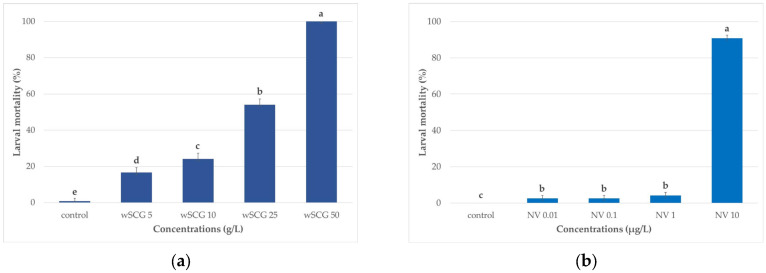
Larval mortality at 72 h for *Aedes* larvae exposed to different concentrations of the specified treatments (n = 30). (**a**) Wet spent coffee grounds (wSCGs); (**b**) novaluron. The different letters above the bars indicate significant differences among the concentrations as determined by Mann–Whitney U test (*p* < 0.05). wSCGs, wet spent coffee grounds at 0, 5, 10, 25, and 50 g/L. NV, novaluron at 0, 0.01, 0.1, 1, and 10 µg/L. The Kruskal–Wallis test revealed significant differences for the wSCGs treatment (df = 4, $x^{2}$ = 18.4 and *p* = 0.001) and the novaluron treatment (df = 4, $x^{2}$ = 15.6 and *p* = 0.004).

**Figure 2:**
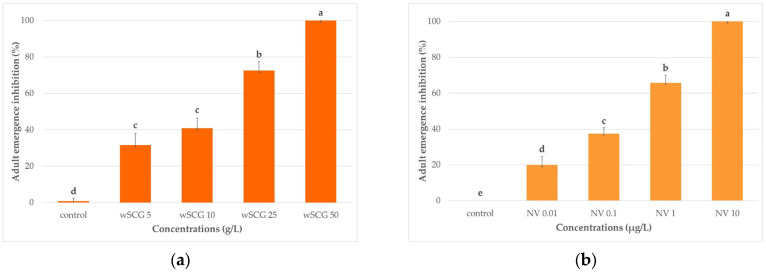
Adult emergence inhibition at 120 h for *Aedes* larvae exposed to different concentrations of the specified treatments (n = 30). (**a**) wSCGs, (**b**) novaluron. The different letters above the bars indicate significant differences among the concentrations as determined by Mann–Whitney U test (*p* < 0.05). The Kruskal–Wallis test revealed significant differences for the wSCGs treatment (df = 4, $x^{2}$ = 18.1 and *p* = 0.001) and the novaluron treatment (df = 4, $x^{2}$ = 18.6 and *p* = 0.001).

After 6 h, larvae exposed to wSCGs at 50 g/L resulted in 22.5 ± 7.4% mortality. At other concentrations, the larvae all remained alive. The wSCGs caused 100% mortality within 48 h of exposure. The wSCGs at 50 g/L also had the highest larval mortality rate at 72 h (Figure 1a), and adult emergence inhibition at 120 h (Figure 2a). 

At 6 and 12 h of exposure, all larvae remained alive in all concentrations of novaluron. At 24 h, the death of larvae began at 0.8 ± 1.7% in 10 µg novaluron/L, whereas the larvae were alive in other concentrations. Novaluron at 10 µg/L (NV 10) had the highest larval mortality in concentration-dependent order in novaluron at 1 (NV 1), 0.1 (NV 0.1), and 0.01 (NV 0.01) µg/L, respectively, at 72 h (Figure 1b), which was similar to the results at 120 h (Figure 2b). Novaluron caused 100% adult emergence inhibition after 120 h of exposure.

The differences in the larval mortality rates of the single specified concentrations of the wSCGs and novaluron were statistically significant (Figure 1a,b). The differences in adult emergence inhibition between the single specified concentrations for the wSCGs and novaluron were also statistically significant (Figure 2a,b).

#### 3.3.2. Synergistic Effects of Sublethal Concentration

The concentrations that cause less than twenty percent larval mortality at 72 h for the stand-alone treatments of wSCGs (5 g/L) and novaluron (0.01, 0.1, and 1 µg/L) were selected to investigate their synergistic effects. At 72 h, 5 g wSCG/L combined with 0.01 (wSCG 5_NV0.01), 0.1 (wSCG 5_NV0.1), and 1 (wSCG 5_NV1) µg novaluron/L resulted in mortality of 40.8 ± 3.2%, 42.5 ± 3.2%, and 46.7 ± 2.7%, respectively. 

At 120 h, the wSCG 5_NV0.01, wSCG 5_NV0.1, and wSCG 5_NV1 resulted in adult emergence inhibition of 53.3 ± 3.8%, 56.7± 2.7%, and 60.8 ± 5.0%, respectively.

**Figure 3:**
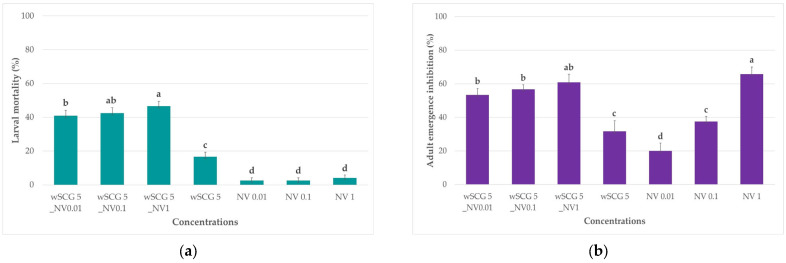
*Aedes* larvae exposed to 5 g wSCG/L combined with different concentrations of novaluron (0.01, 0.1, and 1 µg/L) and compared with those exposed to 5 g wSCG/L or novaluron 0.01, 0.1, and 1 µg/L alone at different time points (n = 30). (**a**) Larval mortality at 72 h. (**b**) Adult emergence inhibition at 120 h. Different letters above the bars indicate significant differences among concentrations as determined by Mann–Whitney U test (*p* < 0.05). The Kruskal–Wallis test showed significant differences for the larval mortality rates at 72 h (df = 6, $x^{2}$ = 24.8 and *p* < 0.001) and the adult emergence inhibition rates at 120 h (df = 6, $x^{2}$ = 24.6 and *p* < 0.001).

The wSCGs at 5 g/L combined with novaluron at concentrations of 0.01, 0.1, and 1 µg/L exhibited higher larval mortality and adult emergence inhibition rates compared to its stand-alone concentrations. The larval mortality rates in this combination are significantly higher than those in the stand-alone treatments (*p* < 0.05), suggesting a potential synergistic effect (Figure 3a,b). 

Overall, the larval mortality and adult emergence inhibition caused by the combination treatment were higher than those of its stand-alone concentrations. At 72 h of exposure, the differences in the larval mortality rates between the combination groups and the sublethal concentrations of wSCGs and novaluron were statistically significant (Figure 3a). At 120 h of exposure, the difference between the adult emergence inhibition of the combination groups and the sublethal concentrations of wSCGs and novaluron were also statistically significant (Figure 3b).

## 4. Discussion

In this study, the effects of wSCGs were compared with those of dSCGs for larval mortality and the adult emergence inhibition of the third-instar larvae of *Ae. aegypti*. The results showed that all larvae died after 72 h of exposure to 50 g/L wSCGs, whereas dSCGs at the same concentration exhibited only 14.2% mortality, indicating wSCGs were more effective than dSCGs. A previous study found that wSCGs left over from coffee capsules brewed strong and light (European coffee) could result in the death of *Ae. aegypti* and *Ae. albopictus* larvae [26]. Furthermore, when the experiment began in the egg stage, wSCGs at concentrations of 50 and 100 mg/mL inhibited the development of *Ae. aegypti* larvae and causing complete death of larvae at the L3 and L2 stages, respectively [15]. Another study using wSCGs to determine the mortality of *Ochlerotatus notoscriptus* larvae found that a concentration of 0.50 g/mL resulted in the highest larval mortality rates (97%) [27]. Several studies using dSCGs have also shown that they can cause mortality and inhibit mosquito larvae emergence into adults. The shade-dried decaffeinated coffee grounds at concentrations of 150 and 200 mg/mL caused 100% mortality of *Ae. aegypti* larvae after exposure for approximately 4–5 days [28]. In addition, three types of dried, roasted arabica coffee grounds (light, medium, and dark) were compared with temephos for their ability to control *Ae. aegypti* larvae. The results showed that using light-roasted dSCGs at 125 mg/mL had the highest efficacy, causing almost 100% mortality after 24 h of exposure. Although not as effective as temephos, light-roasted coffee grounds were also found to be effective in the death of mosquito larvae [29]. 

We found total phenolic compounds, caffeic acid, coumaric acid, protocatechuic acid, vanillic acid, and total flavonoid compounds in SCGs, but the levels were higher in the wSCGs compared with those of dSCGs. These chemical compounds were found to affect insect survival and inhibit their development into adults. Therefore, wSCGs, which had more phenolic and flavonoid compounds, could cause higher larval mortality and adult emergence inhibition in *Ae. aegypti* larvae. The SCGs contain phenolic compounds (chlorogenic acids, caffeic acid, tannic acid, coumaric acid, protocatechuic acid, etc.), alkaloid compounds (caffeine and trigonelline), and diterpenes (cafestol and kahweol) [30,31,32]. Alkaloids, flavonoids, and tannins can block parasympathetic nerves in the insect nervous systems. These substances can inhibit the absorption of food and decrease the action of digestive enzymes when consumed by the larvae, leading to a slow death. Additionally, the bitter flavor of SCGs may irritate the larvae when consumed [33]. Phenolic and flavonoid compounds are plant secondary metabolites known as antioxidants that can affect insect mortality, development, and physiology [34]. Phenolic compounds affect the midguts of insects, where they act as pro-oxidants and induce oxidative stress and reactive oxygen species [35]. For example, ingestion of caffeic acid by *Spodoptera littoralis* larvae can reduce α-amylase activity, protease activity, and the number of immunoreactive cells in the midgut. After feeding caffeic acid to *S. littoralis* for 10 days, the survival rate was found to be decreased by 80–95% [36]. In addition, caffeic acid is involved in inhibiting digestive proteases and affecting growth, thereby reducing the survival rate by 50–80% in *Helicoverpa armigera* [37]. Other studies on *H. armigera* have shown that ingesting chlorogenic acid, caffeic acid, and protocatechuic acid at 1000 ppm correlated to 42.50%, 37.20%, and 34.50% mortality rates, respectively. Moreover, these phenolics caused *H. armigera* to have low larval weights by decreasing protease and trypsin activities in the midgut [38]. Vanillic acid, 4-hydroxy benzoic acid, and cinnamic acid extract from *Rosmarinus officinalis* leaves cause an average mortality rate of 81.67% in *Tribolium castaneum* adults [39]. Coumaric acid and resveratrol can reduce larval weights, delaying the pupation of *Spodoptera litura* and *Amsacta albistriga* larvae. In contrast, these compounds increase the antioxidant enzymes of these insects [40]. Caffeine can affect the physiology of insects by altering their metabolism and gas transport patterns, which also decreases their survival ability [41]. A study on *Ae. aegypti* revealed that larvae exposed to caffeine at 2 mg/mL had 100% larval mortality in 6 days after exposure (LT_50_, 3 days after exposure) [28]. Caffeine at 1.5% and 2.0% was mixed with wheat kernels, which lead to high mortality rates in *Sitophilus oryzae* adults (54.43% and 55.21%, respectively) after 5 days of exposure, and at the end of the experiment (2 weeks), no adults had survived [42].

Novaluron is an IGR, which is normally used in agriculture to control pests. The WHOPES suggested that 10–50 µg novaluron/L could be applied to the temporary habitats of mosquitoes as a larvicide [21]. The results of this study support the recommended dose of WHOPES, as there was 100% larval mortality for *Ae. aegypti* when exposed to 10 µg/L for 120 h. Novaluron has been found to induce larval mortality and inhibit adult emergence in several mosquito species, such as *Ae. aegypti* [43,44,45], *Ae. albopictus*, *Anopheles albimanus*, *Anopheles pseudopunctipennis*, *Culex quinquefasciatus* [44], and *Culex pipiens* [46]. Field-based studies in Thailand have shown that using 0.05–1.00 mg novaluron/L in clay jars can inhibit the emergence of *Ae. aegypti* larvae by 86–96% for 190 days [43]. Furthermore, novaluron at 10 mg a.i./m^2^ was found to reduce 90–100% of the immature *Cx. quinquefasciatus* mosquitoes for 3–7 weeks in polluted water [47]; the same concentration was also found to reduce the prevalence by 50% within 24 h, as it inhibited the development of *Cx. quinquefasciatus* larvae into adults, and the effect lasted for 4 weeks [48]. However, negative effects on fishes, aquatic plants, and terrestrial plants from the application of novaluron were not reported [47,48].

The combination of insecticides and plant extracts has been examined in previous studies. The larvicidal and synergistic effects of organophosphates with extracts of plants, such as *Vinca rosea*, *Leucas aspera*, and *Clerodendrum inerme*, were revealed against *Cx. quinquefasciatus*, *Anopheles stephensi*, and *Ae. aegypti* [49]. Permethrin combined with essential oils (from *Cyperus rotundus* and *Alpinia galanga*) against *Ae. aegypti* adults were found to have a synergistic effect (synergism ratio of 6.28 and 4.00, respectively) [50]. In this study, it was found that the sublethal concentration combination of both wSCGs and novaluron increased larval mortality and inhibited adult emergence more effectively compared to the sublethal concentration of stand-alone treatments with either wSCGs or novaluron, at every observation point. This suggests that there is a synergistic effect at sublethal concentrations when wSCGs and novaluron are combined. Exposure to sublethal-dose wSCGs causes a decrease in digestive enzymes and damages the midgut, resulting in the weakness of larvae but not death, which is found with exposure to high concentrations. Novaluron is a chitin synthesis inhibitor, with a role in larval stage development. However, the death of larvae occurred at long-term exposures and may be caused by the accumulation of the low concentrations of wSCGs and novaluron. These combinations could thus be used to effectively control mosquito larvae in the future.

The popularity of drinking coffee worldwide has led to environmental concerns about its waste (SCGs), which includes caffeine and phenolic compounds, of which caffeine is the most predominant. Caffeine pollution in the environment, however, is not only from SCGs but also from numerous beverages and the pharmaceutical industry. It is also excreted by humans in their urine after consumption and released from households into wastewater treatment plants (WWTP) [51,52]. Caffeine contamination has been reported in the rivers, WWTP and seawater of many countries, such as Brazil [53,54], China [55], Japan [56], Greece [57], Taiwan [58], Spain [59], and the USA [60]. Several studies have also reported the detection of caffeine in the tissues of aquatic animals [61,62], but the toxicity thresholds differ depending on the species [63]. Caffeinated waters are generally detected at low levels (varying from ng/L to µg/L). Caffeine degradation gradually occurs by photolysis [64] and biodegradation [65], with a half-life of around 1.50 days in water [64]. Therefore, long-term studies of the toxicity of caffeine in the environment are also required [63], along with the optimization of the wastewater treatment system for more efficient caffeine removal [66]. Novaluron has low solubility in water (3 µg/L) and slow hydrolysis and photolysis rates with a half-life of 101 days at 25 °C, which indicates that novaluron is unlikely to be degraded in the environment. Novaluron has low vapor pressure, which results in negligible volatilization from soil or plant surfaces [20]. Sensitivity to novaluron has been reported in *Daphnia* and mayfly, but it has a low toxicity to mammals (rats: LD_50_ ≥ 5000 mg/kg bw), birds (bobwhite quail: LD_50_ > 2000 mg/kg), fish (rainbow trout: LC_50_ ≥ 1.00 mg/L), honey bees (LD_50_ > 100 µg/bee), earthworms (LC_50_ = 1000 ppm), soil microflora, and aquatic plants [20]. However, a study has found that exposure to novaluron can result in pathological changes to the gill and liver of the fish *Labeo rohita* [67]. In addition, studies have shown that novaluron reduces the weight of the cuticle, the percentage of chitin in the cuticle, and the thickness of the old and new cuticles in the shrimp *Palaemon adspersus*, thus affecting their growth [68,69]. It is thus recommended that wSCGs in combination with novaluron could be used to control mosquitoes in breeding sites in waterlogged areas that are not habitats for aquatic animals in the house, such as flower vases, cabinet stands, and plant pot trays. The suggested minimal effective dose of the combination is wSCGs 5 g/L combined with novaluron 0.01 µg/L.

## 5. Conclusions

The results of this study have shown that wSCGs have higher efficacy on larval mortality and adult emergence inhibition of *Ae. aegypti* than those of dSCGs. A concentration of 50 g/L of wSCGs and 10 µg/L of novaluron proved to be effective for larval mortality and inhibition of adult emergence in *Ae. aegypti*. The combination of sublethal concentrations of wSCGs and novaluron revealed synergistic effects. The results indicate that the combination of wSCGs and novaluron could be utilized as an alternative method for mosquito control. The effectiveness of this combined treatment should be further evaluated in other mosquito species, as well as in field-based studies.

## Figures and Tables

**Table 1:** Chemical analysis of the dry weight of wet spent coffee grounds (wSCGs) and dry spent coffee grounds (dSCGs).

Phytochemicals	Chemicals	Units	wSCGs	dSCGs
Alkaloid compound	Caffeine	%*w*/*w* **	0.2	0.4
Flavonoid compounds	Total flavonoid compounds	mg/g	7.3 ± 0.1	1.1 ± 0.1
Phenolic compounds	Total phenolic compounds	mg/g	50.7 ± 0.9	5.2 ± 0.1
	Caffeic acid	µg/g	329.7 ± 0.4	8.6 ± 2.3
	Coumaric acid	µg/g	120.8 ± 3.1	5.8 ± 0.3
	Protocatechuic acid	µg/g	41.1 ± 30.9	9.1 ± 3.0
	Vanillic acid	µg/g	5597.0 ± 213.8	770.3 ± 22.5
	Ferulic acid	µg/g	ND *	ND *
	Gallic acid	µg/g	ND *	ND *
	Sinapic acid	µg/g	ND *	ND *

* ND: Not detected; ** %*w*/*w* (%weight per weight): weight of a caffeine as a percentage of a total SCGs in dry weight.

**Table 2:** The comparison of larval mortality rates at 72 h between different concentrations of wSCGs and dSCGs (30 larvae/test).

Times (h)	Treatments	Larval Mortality Rates ($\bar{x}$ ± SD)	Treatments	Larval Mortality Rates ($\bar{x}$ ± SD)	U	*p*
72	control	0.8 ± 1.7	control	0.8 ± 1.7	8.0	1.000
	wSCG 5	16.7 ± 2.7	dSCG 5	8.3 ± 1.9	0.0	0.019
	wSCG 10	24.2 ± 3.2	dSCG 10	10.8 ± 3.2	0.0	0.019
	wSCG 25	54.2 ± 3.2	dSCG 25	14.2 ± 4.2	0.0	0.019
	wSCG 50	100	dSCG 50	14.2 ± 5.0	0.0	0.013

**Table 3:** The comparison of adult emergence inhibition rates at 120 h between different concentrations of wSCGs and dSCGs (30 larvae/test).

Times (h)	Treatments	Adult Emergence Inhibition Rates ($\bar{x}$ ± SD)	Treatments	Adult Emergence Inhibition Rates ($\bar{x}$ ± SD)	U	*p*
120	control	0.8 ± 1.7	control	0.8 ± 1.7	8.0	1.000
	wSCG 5	31.7 ± 6.4	dSCG 5	14.2 ± 3.2	0.0	0.019
	wSCG 10	40.8 ± 5.7	dSCG 10	16.7 ± 6.7	0.0	0.018
	wSCG 25	72.5 ± 5.0	dSCG 25	24.2 ± 1.7	0.0	0.015
	wSCG 50	100	dSCG 50	25.0 ± 7.9	0.0	0.013

## Data Availability

The data presented in this study are available within the article.
